# Effect of full flavor and denicotinized cigarettes exposure on the brain microvascular endothelium: a microarray-based gene expression study using a human immortalized BBB endothelial cell line

**DOI:** 10.1186/s12868-015-0173-3

**Published:** 2015-06-23

**Authors:** Pooja Naik, Ravi K Sajja, Shikha Prasad, Luca Cucullo

**Affiliations:** Department of Pharmaceutical Sciences, School of Pharmacy, Texas Tech University Health Sciences Center, 1300 S. Coulter Street, Amarillo, TX 79106 USA; Center for Blood Brain Barrier Research, Texas Tech University Health Sciences Center, Amarillo, TX 79106 USA

**Keywords:** Environment, Endothelium, Blood brain barrier, Inflammation, Transcriptomics, Alternative

## Abstract

**Background:**

Tobacco smoke (TS) toxicity to the brain microvasculature is still an understudied area till date. NF-E2 related factor (Nrf2) is a key transcription factor responsible for activating the antioxidant response element (ARE) genes following an oxidative insult. Till date, several studies targeting the blood brain barrier (BBB) have shown some protective role of Nrf2 in ischemia–reperfusion (IR) injury, however, its functional role in chronic smokers subjected to a life-long oxidative stress has never been addressed. This is of crucial importance since smokers have a much higher risk for cerebrovascular stroke and tobacco smoke exposure has been clearly shown to enhance BBB damage following an ischemia/reperfusion injury. Thus, the goal of our study was to investigate the defense pathways activated at the BBB endothelial level by TS exposure. Specifically we focused on Nrf2 and nuclear factor kappa-light-chain-enhancer of activated B signaling response (NF-κβ) as the central protective mechanisms related to oxidative insult.

**Results:**

With the exception of Nicotine, both full flavor (3R4F) and decotinized (ULN) cigarettes activated Nrf2 and NFκβ pathways in hCMEC/D3 endothelial cells. Several detoxification and anti-oxidant genes including downstream products were also activated including NAD(P)H dehydrogenase quinone 1 (NQO-1), heme oxygenase-1 (HMOX-1), catalytic and modifier subunits of glutamate-cysteine ligase (GCL), solute carrier-SLC7A11). Gene expression levels of cytochrome P450s (CYP2S1 and CYP51A1) and efflux transporters P-glycoprotein (P-gp) and multi-drug resistance protein-4 (MRP4) were also enhanced. Increase of P-gp functional activity and depletion of GSH were also observed. Strikingly, toxicity of denicotinized (“reduced exposure”) cigarettes was equivalent to 3R4F (or worse).

**Conclusions:**

This study provides a detailed analysis of Nrf2-related cytoprotective mechanisms activated in response to 3R4F and ULN-derived TS exposure correlating the results with their oxidative and inflammatory potential. Toxicants present in soluble cigarette smoke extracts (CSE) and not nicotine seem to be the primary determinant of vascular toxicity. In this respect our results from this and previous studies suggest that chronic TS exposure can overcome Nrf2 and NFκB-p65 dependent cytoprotective mechanisms of the brain microvascular endothelium possibly leading to BBB impairment and loss of BBB integrity.

**Electronic supplementary material:**

The online version of this article (doi:10.1186/s12868-015-0173-3) contains supplementary material, which is available to authorized users.

## Background

Tobacco smoke (TS) is the leading cause of premature preventable death worldwide. Smoking kills nearly 6 million over the world with 480,000 deaths in United States alone [1, 2]. Smoking attributable deaths have been largely associated to the pathogenesis of several complications such as cancer, ischemic heart disease and chronic obstructive pulmonary disease (COPD) [1]. However, equally significant but often neglected are the effects of chronic smoking on the onset and progression of several neurological and neurovascular complications such as Alzheimer’s disease, multiple sclerosis and cerebral stroke [3–5]. Although epidemiological studies establish cigarette smoking as a major risk factor for the onset and progression of these diseases; till date the precise mechanisms underlying these irreversible deleterious changes to the brain and brain vasculatures are poorly understood.

Tobacco smoke consist of several thousand toxic compounds (including nicotine) capable of inducing oxidative stress and vascular inflammation triggering several pathophysiological changes in peripheral vasculatures [6, 7]. Specifically to the blood brain barrier (BBB); we have clearly demonstrated that (1) TS can degrade membrane expression of essential tight junction (TJ) proteins such as ZO-1 and occludin; (2) it can induce a vascular inflammatory response in BBB endothelial cells (ECs) via release of Interleukin-6 (IL-6) and matrix metalloproteinase-2 (MMP-2); (3) it can up-regulate vascular adhesion molecules such as vascular adhesion molecule-1 (VCAM-1) and platelet endothelial cell adhesion molecule-1 (PECAM-1). All together, the data clearly suggest that TS can severely impair and compromise BBB integrity and function [8–10].

Moreover, we have also clearly demonstrated that oxidative stress is one of the cardinal determinants of TS induced BBB toxicity where the extent of BBB damage positively correlates with the oxidative capacity of the TS product tested. Several reduced exposure ‘light’ versions of tobacco products are currently available in market that claim to be safe solely on the reduction of nicotine or a few selected toxicants. Profiling of TS showed a significant release of reactive oxygen (ROS), hydrogen peroxide content and reactive nitrogen species (RNS) in the nicotine-free (NF, “reduced-exposure” brand) and ultralow nicotine products along with the full flavor products. As the level of tar, H_2_O_2_ and nitric oxide content increased; TS induced BBB toxicity increased proportionally along with the level of pro-inflammatory activity and oxidative stress.

Thus we further investigated the role of oxidative stress and anti-oxidant mechanisms (such as NF-E2 related factor—Nrf2—signaling pathway) activated at the BBB endothelium in response to TS exposure. This is one of the main protective mechanisms triggered to counteract oxidative insult such as those elicited by TS [11].

Although microarray based gene expression studies investigating TS induced global changes in cellular transcriptome have been previously reported in human bronchial epithelium, oral mucosa and peripheral immune cells [12–15]; the effect of TS on BBB endothelium has only be marginally addressed and even more so at the level of gene expression and transcription. Moreover, a comprehensive study focussing on TS activated anti-oxidant mechanisms at the BBB is still lacking. In this study we addressed this critical issue using human BBB endothelial cells (hCMEC/D3 cell line) [16] chronically exposed to various TS related products over a period of 24 h. Nrf2 expression and its downstream pathway targets were also assessed and correlated with respect to oxidative and/or inflammatory potential of the specific tobacco product.

For this purpose, we performed a side-by-side comparative analysis of antioxidant mechanisms triggered by nicotine exposure against soluble cigarette smoke extracts (CSE) from: (1) regular full flavor cigarette (3R4F) and (2) reduced exposure ultra-low nicotine cigarette (ULN). About 43,000 gene targets were screened in a microarray based gene expression studies. Positive hits from the gene array data were investigated further using real time PCR (mRNA) and western blot analysis (proteins expression level) as well as ad hoc functional studies. In summary, our work provides an in depth detailed analysis of the BBB counteractive cellular mechanisms triggered by realistic exposure to TS.

## Results

### CSE increases oxidative stress load and Nrf2 nuclear translocation in BBB endothelium

**Figure 1:**
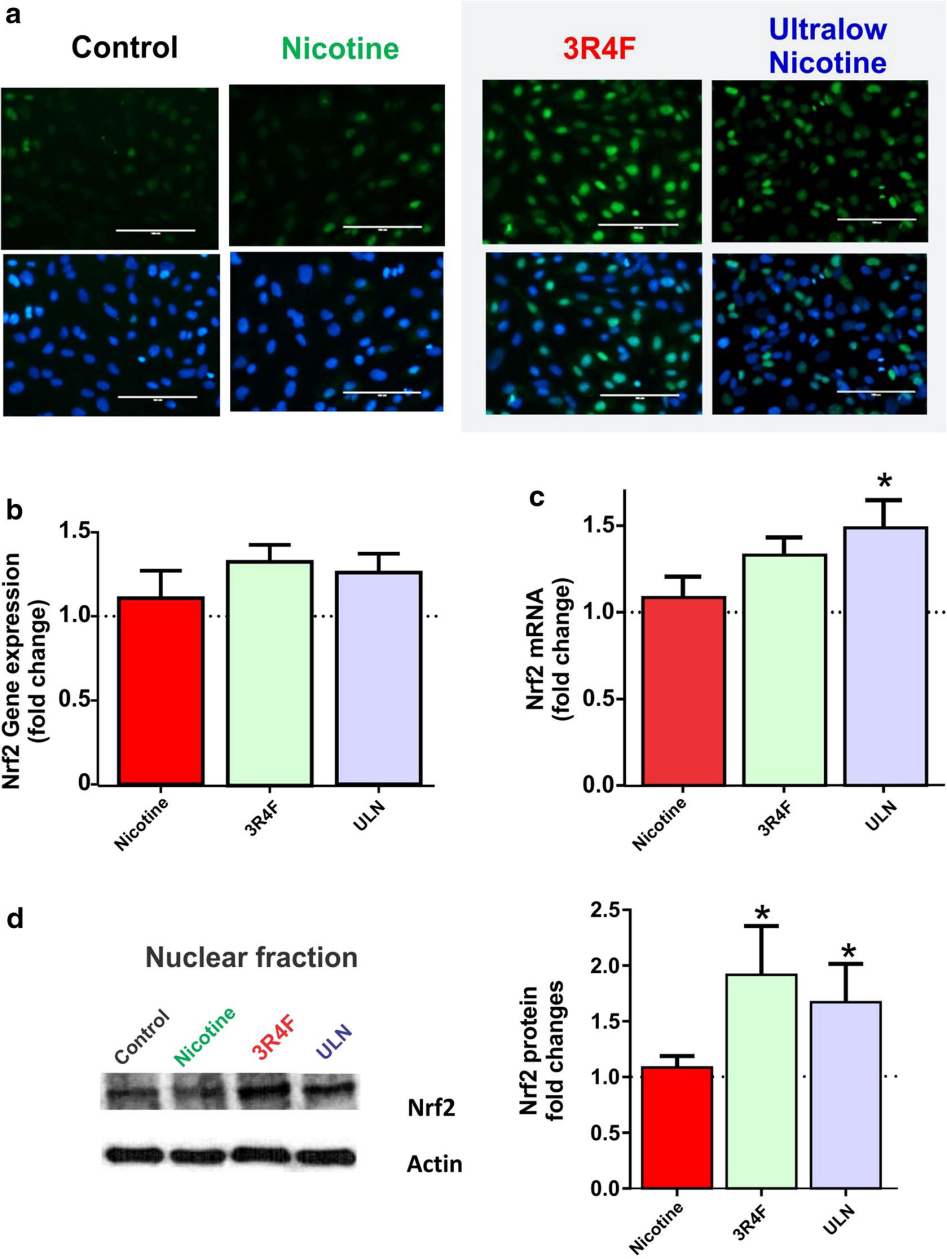
CSE exposure potentiates oxidative stress responses in BBB endothelial cells. Briefly, confluent hCMEC/D3 cell monolayers were exposed to media containing nicotine only (100 μg/mL) or CSEs from full flavor (3R4F) or ULN tobacco products containing nicotine equivalent to 100 ng/mL). Fresh media without nicotine or mainstream TS served as controls. **a** Immunofluorescence analysis of cellular ROS levels (an indicator of oxidative stress load) were determined by using CellROX^®^ Green reagent following exposure to CSE or nicotine treatment (3 h); **b** gene array based analysis of Nrf2 gene expression changes was determined after control, nicotine or TS exposure (n = 6); **c** Nrf2 mRNA expression was quantified by RT-PCR using primer specific sequences (n = 4); **d** Nrf2 expression and nuclear translocation following exposure to different conditions at early time point of 8 h as analyzed by western blotting. Representative western blots were shown with actin as a loading control. Data were expressed as mean ± SEM (fold change over control; n = 4 biological replicates). *p < 0.05 vs. control.

Main and side stream TS contain high levels of toxic reactive aldehydes and pro-oxidants that are shown to destabilize the cellular redox balance. We assessed the impact of TS generated from different tobacco products, on cellular ROS levels (oxidative stress load) in BBB endothelium using CellROX^®^ Green Reagent. As shown in Figure 1a, exposure to CSE from both full flavor (3R4F) and ULN cigarettes rapidly increased endothelial ROS (as early as 3 h post-exposure), with greater intensity of fluorescence, compared to control. In comparison, exposure to nicotine elicited only a mild oxidative response (Figure 1a).

Next, we determined the effects of TS on endothelial Nrf2 expression and its nuclear translocation (Figure 1b–d). Nrf2 is a redox-sensitive transcription factor and a master regulator of cellular redox homeostasis. It has been demonstrated that increased levels of ROS trigger the activation and subsequent nuclear translocation of Nrf2. This ultimately triggers the expression/activation of various molecular networks primarily involved in cellular cytoprotection against oxidative and inflammatory stress [11, 17]. To this end, our data showed modest increase in Nrf2 gene expression levels in response to CSE from 3R4F and ULN cigarettes (Figure 1b). However, Nrf2 transcription assessed by RT-PCR was significant in BBB endothelial cells exposed to ULN-derived CSE (p < 0.05; compared to control) but not in cultures exposed to CSE derived from 3R4F cigarettes (Figure 1c) Interestingly, Nrf2 nuclear translocation (Figure 1d) was statistically significant in BBB ECs exposed to either ULN and 3R4F cigarette extracts. Nicotine exposure did not affect Nrf2 gene expression (Figure 1b) nor Nrf2 transcription (Figure 1c) or its nuclear translocation (Figure 1d). This further supports its relatively modest impact on cellular oxidative stress levels observed in Figure 1a.

### CSE up-regulates Nrf2-ARE dependent phase I detoxification genes

**Figure 2:**
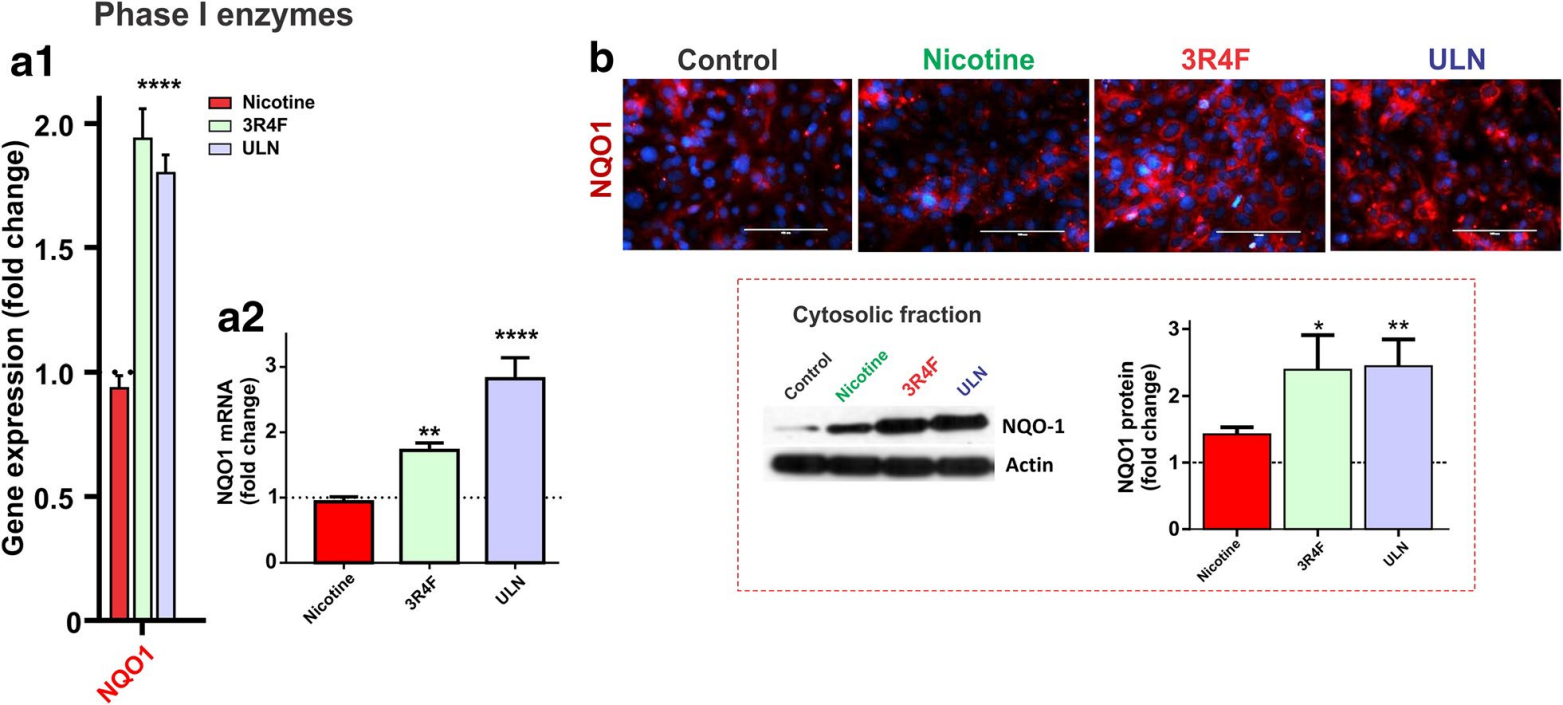
Effects of CSE on Phase I detoxification genes. HCMEC/D3 cells were exposed to nicotine or CSE from different products (see “Methods”). Effects of CSE and nicotine on NQO-1 mRNA and protein expression were characterized by: **a1** gene array analysis; **a2** real-time RT-PCR; **b** immunofluorescence and western blot analyses of NQO-1 protein. Representative western blots were shown with actin as a loading control. Data were expressed as mean ± SEM (fold change over control). *p < 0.05, **p < 0.01, ****p < 0.0001 vs. control. n = 6/biological replicates/condition.

As shown in Figure 2a, both microarray and RT-PCR analyses revealed a significant induction of NQO1 gene (a major enzyme of Phase I detoxification) both by 3R4F and ULN products (p < 0.01 and p < 0.0001 vs control fold change respectively), but not nicotine. Accordingly, exposure to 3R4F and ULN upregulated NQO1 protein expression as demonstrated by immunofluorescence and western blotting (Figure 2b; p < 0.05 and p < 0.01 for 3R4F and ULN, respectively). Notably, the up-regulation of NQO1 expression by ULN was comparable to that achieved by 3R4F.

### CSE exposure augments transcription of Phase I cytochrome P450s detoxification genes

**Figure 3:**
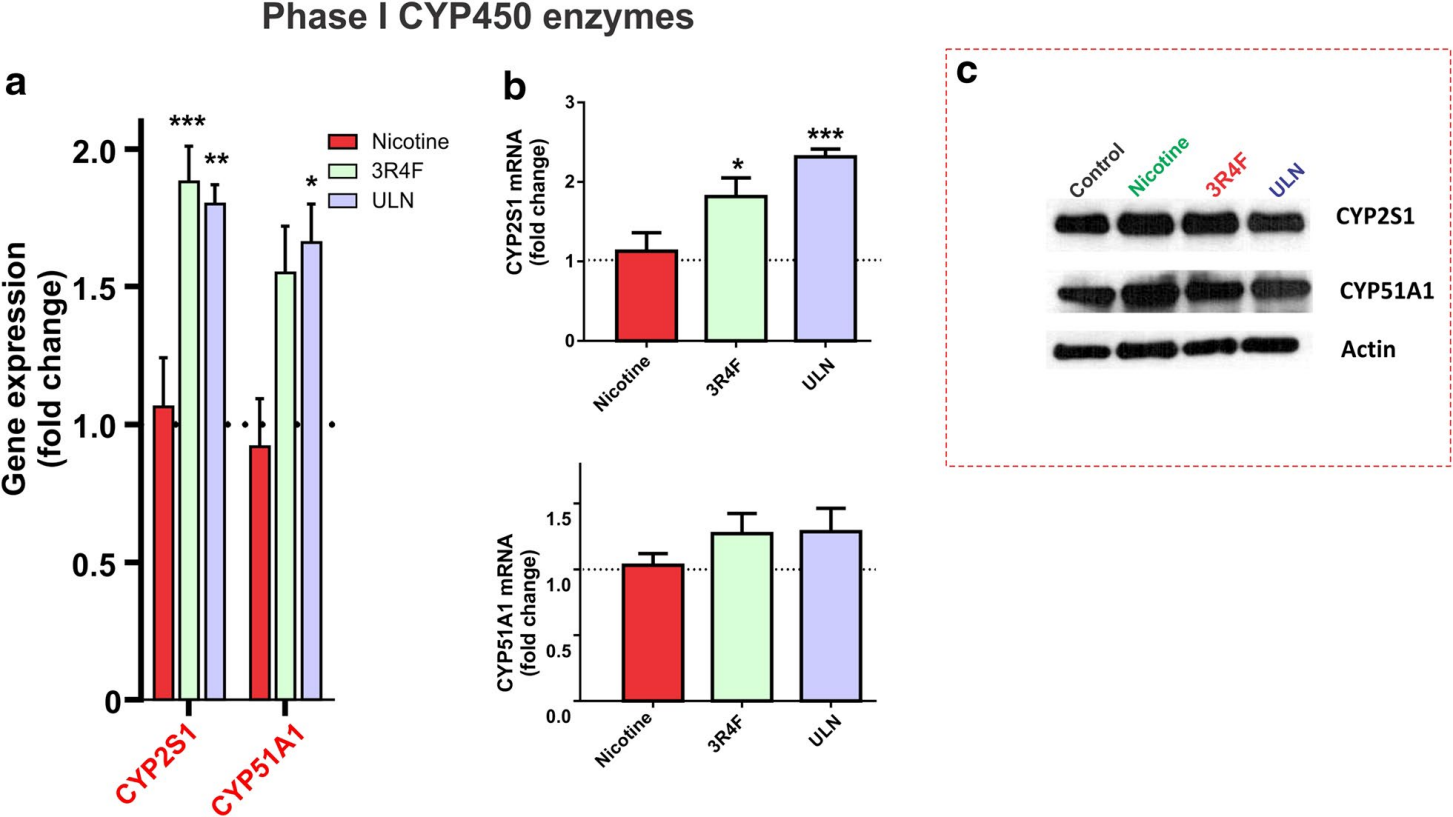
Effects of CSE on Phase I cytochrome P450s detoxification genes. As mentioned earlier, hCMEC/D3 cell monolayers were exposed to nicotine (100 ng/mL) or CSE derived from 3R4F or ULN cigarettes. Following exposure for 24 h, expression of CYPs-CYP2S1 and CYP51A1 was analyzed by **a** DNA microarray, **b** real time PCR for mRNA; and **c** protein expression was analyzed by western blotting. Representative western blots were shown with actin as a loading control; data were expressed as mean ± SEM (fold change over control). *p < 0.05, **p < 0.01, ***p < 0.001 vs. control. n = 6 biological replicates/condition.

Cytochrome P450 (CYP450) family related enzymes provide additional layer of counter-regulatory anti-oxidant response through metabolism-based detoxification of toxic chemicals [18]. Although there is no specific report of CYP51A1 with respect to smoke toxicants, CYP2S1 has been reported to scavenge xenobiotics especially carcinogens present in TS [19]. As shown in Figure 3a, we observed a striking up-regulation in the gene expression of the CYP enzymes CYP2S1 (Figure 3a, p < 0.001 and p < 0.01 vs. control, for 3R4F and ULN respectively) which was also confirmed by RT-PCR (Figure 3b, p < 0.05 and p < 0.001 vs. control for 3R4F and ULN respectively). CYP51A1 gene expression was also up-regulated however; the measured changes were statistically significant only for BBB endothelial cultures exposed to ULN but not for 3R4F-derived CSE (Figure 3a). Corresponding RT-PCR measurements of the gene transcription showed only a modest increased but not to a significant level in either case. Note also that none of the changes in either gene expression or gene transcription for both enzymes failed to translate in a corresponding increase in protein expression (see Figure 3c). No effect at any level (gene expression, transcription and translation) was observed in response to nicotine exposure.

### CSE alters the efflux activity but not the expression of BBB P-glycoprotein

**Figure 4:**
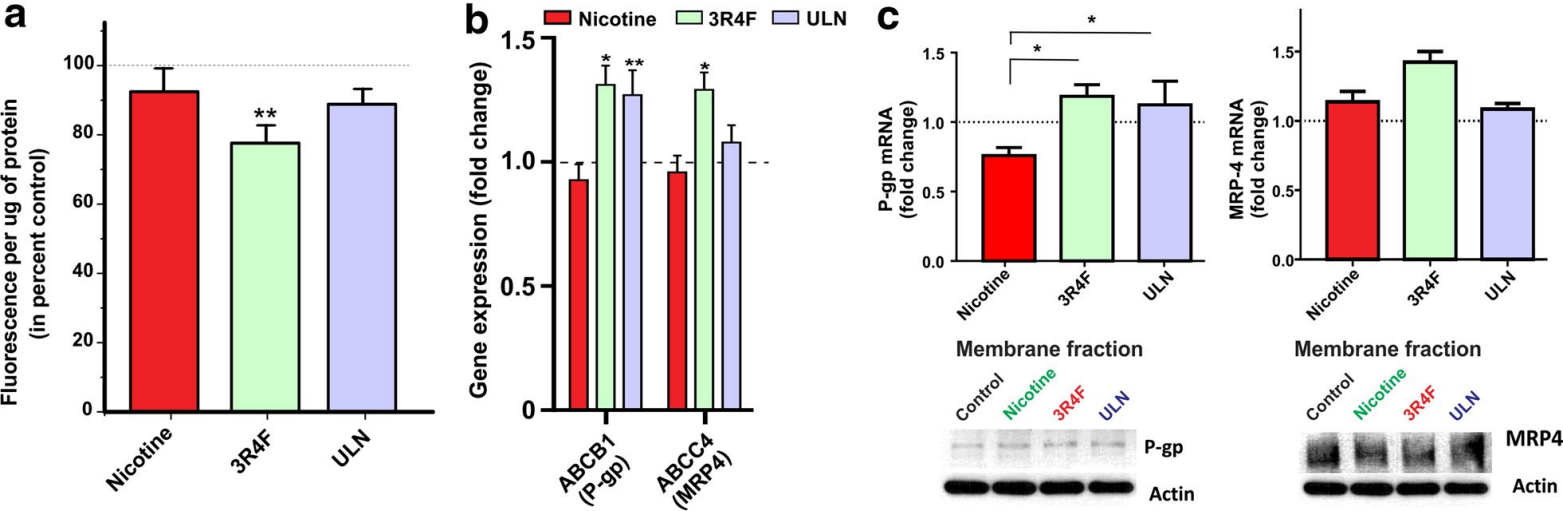
Effects of CSE on ABC efflux transporter expression and functionality in hCMEC/D3 cell line. Cells were exposed to nicotine (100 ng/mL) or CSE derived from 3R4F or ULN cigarettes. **a** P-gp efflux activity was determined by intracellular accumulation of rhodamine123 (a P-gp substrate) efflux, as an indirect correlate of P-gp activity n = 3/condition and replicated twice. **b** Transcriptome analysis of ABC efflux transporters, ABCB1 (P-gp) and ABCC4 (MRP4) following treatment (n = 6 biological replicates); **c** RT-PCR analysis of mRNA expression of P-gp and ABCC4 in hCMEC/D3 cells (n = 6 biological replicates); western blot analysis of transporter protein expression (n = 6 biological replicates). Representative western blots were shown with actin as a loading control. Data were expressed as mean ± SEM (fold change over control). *p < 0.05, **p < 0.01, vs. control.

Blood brain barrier endothelial cells are highly enriched with polarized expression of ATP-binding cassette superfamily of drug efflux transporters such as P-glycoprotein (P-gp) that prevent the brain penetration and accumulation of toxic substances including xenobiotics. Previous studies have shown that Nrf2 activation induces the transcription of major drug efflux transporters [11]. As shown in Figure 4, microarray analysis revealed a significant up-regulation of two major ABC efflux transporters, P-glycoprotein (P-gp; ABCB1) and multidrug resistant protein-4 (MRP-4; ABCC4; p < 0.05, vs. control) in response to 3R4F exposure. The biological effect is consistent with a marked increase in rhodamine123 efflux (p < 0.05, vs. control) used as a measure of P-gp functional activity. CSE from ULN also increased the gene expression of P-gp (p < 0.01 vs. control), but not that of MRP4 (Figure 4b). However, RT-PCR and western blotting analysis of the cellular membrane fractions did not show a corresponding increase in gene transcription and/or protein expression levels for either of these transporters (Figure 4c). Interestingly, when compared to nicotine, TS did produce a statistically significant increase of P-gp transcription when compared to nicotine since surprisingly nicotine caused a modest reduction in P-gp transcription when compared to controls.

### CSE potentiates synthesis and activity of various antioxidants in BBB endothelial cells

**Figure 5:**
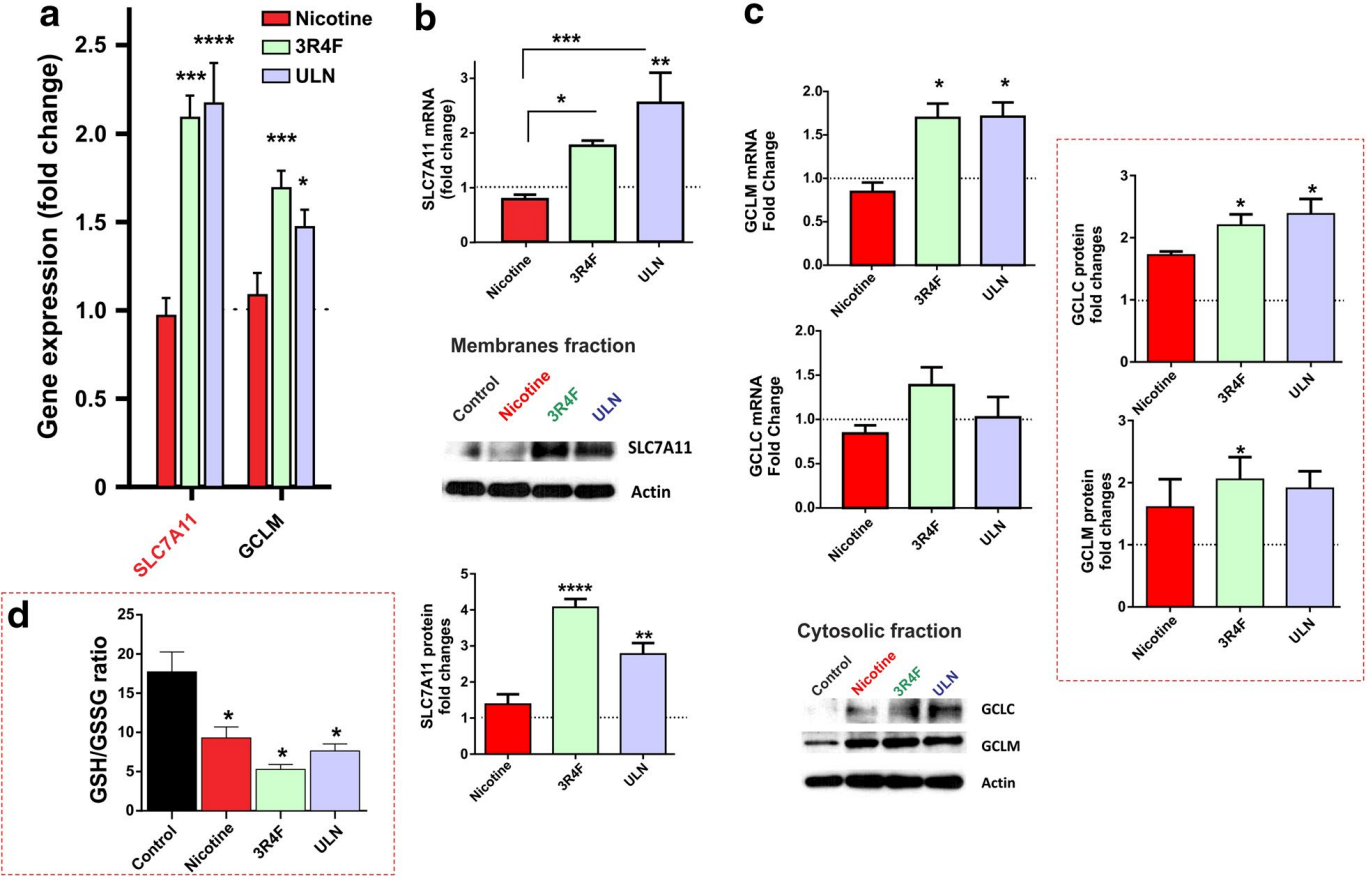
Effects of CSE on GSH based antioxidant system. **a** Microarray studies revealed an up-regulation in gene expression of two major genes involved in GSH synthesis, SLC7A11 and GCLM; **b** quantitative assessment using real-time PCR and western blot analyses confirm microarray analysis. **c** mRNA expression of modifier and catalytic subunits of GLC and protein expression of GCLC and GCLM following CSE exposure derived from 3R4F and ULN products including nicotine. Representative western blots were shown with actin as a loading control; **d** GSH/GSSG ratio was determined using Thiol green reagent, as mentioned in the methods section. Data were expressed as mean ± SEM (fold change over control). *p < 0.05, **p < 0.01, ***p < 0.001, ****p < 0.0001 vs. control. n = 6 biological replicates/condition.

The next major component of the Nrf2-ARE pathway is the anti-oxidant system. In this study, we compared the effects of CSE derived from conventional and reduced exposure products on the genes involved in synthesis of major anti-oxidant glutathione (GSH), such as glutathione cysteine ligase and SLC7A11 [20]. Genome wide transcriptional profiling revealed an amplified gene expression of GCL-modifier unit (GCLM) and SLC7A11 in BBB endothelial cells exposed to CSE from 3R4F and ULN (Figure 5a). These results were further validated by RT-PCR analysis indicating a potential up-regulation of SLC7A11 (Figure 5b, p < 0.05 and p < 0.01 vs. control) and GCLM transcription (Figure 5c, p < 0.05 vs. control) by 3R4F and ULN smoke extracts, respectively. Moreover, alterations in the protein expression of SLC7A11 and GCLM followed a similar trend with significant increase upon exposure to CSE derived from both products (Figure 5b, c). In addition, 3R4F but not ULN moderately increased gene transcription of GCL-catalytic subunit (GCLC), while GCLC protein expression was up-regulated by both products to a similar extent (Figure 5c). By contrast, nicotine did not produced any significant alterations in GCLM and SLC7A11 gene expression, although we observed a marked increase in the cytoplasmic expression of the catalytic and modifier subunits of GCL following nicotine exposure, as shown by western blotting (Figure 5c). Importantly, all the tested products (including nicotine) reduced the turn-over rate of cellular GSH, as demonstrated by the GSH/GSSG ratio (Figure 5d, p < 0.05). This clearly suggests a depletion of cellular antioxidant protection against incumbent oxidative stress load caused by the exposure to smoke extracts and nicotine (in smaller measure).

**Figure 6:**
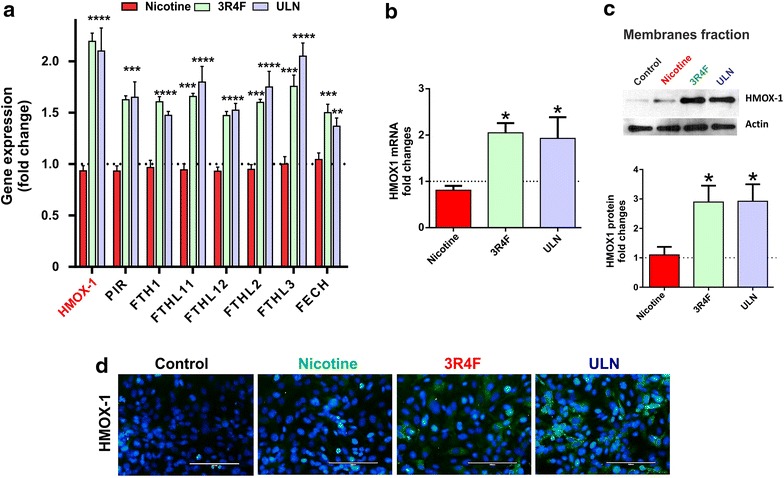
Effects of CSE exposure (24 h) on BBB endothelial gene and protein expression of HMOX1. **a** Several genes belonging to HMOX-1 and heme recycling were up-regulated following CSE exposure of TS products. Nicotine did not produce any change in these genes; **b** real time RT-PCR showed up-regulation of HMOX-1 protein expression following exposure to full flavor, and ULN CSEs; **c** western blot analyses of cellular membrane fractions corroborate with mRNA changes showing statistically significant up-regulation in protein expression; representative western blots were shown with actin as a loading control; **d** immunofluorescence analysis of HMOX1 expression. Data were expressed as mean ± SEM (fold change over control). *p < 0.05, **p < 0.01, ***p < 0.001, ****p < 0.0001 vs. control. n = 6 biological replicates/condition.

Continuing on the same path, we assessed the effects of nicotine, 3R4F and ULN exposure on gene expression, transcription and translation of heme oxygenase (HMOX-1). HMOX-1 is also a component of the cellular antioxidant cytoprotective mechanisms along with other molecular networks (including ferritin and pirin involved in iron/metal sequestration) [21]. As shown in Figure 6a, transcriptome analysis revealed a significant increase in the gene expression levels of HMOX-1 and other anti-oxidant molecules in response to 3R4F and ULN exposure. Results were corroborated by RT-PCR and western blot analyses in line with gene expression data as shown in Figure 6b, c respectively (p < 0.05, vs. control). Immunofluorescence analysis of the BBB endothelial monolayers further supports these observations (Figure 6d).

### CSE increases NF-κβ expression and nuclear translocation

**Figure 7:**
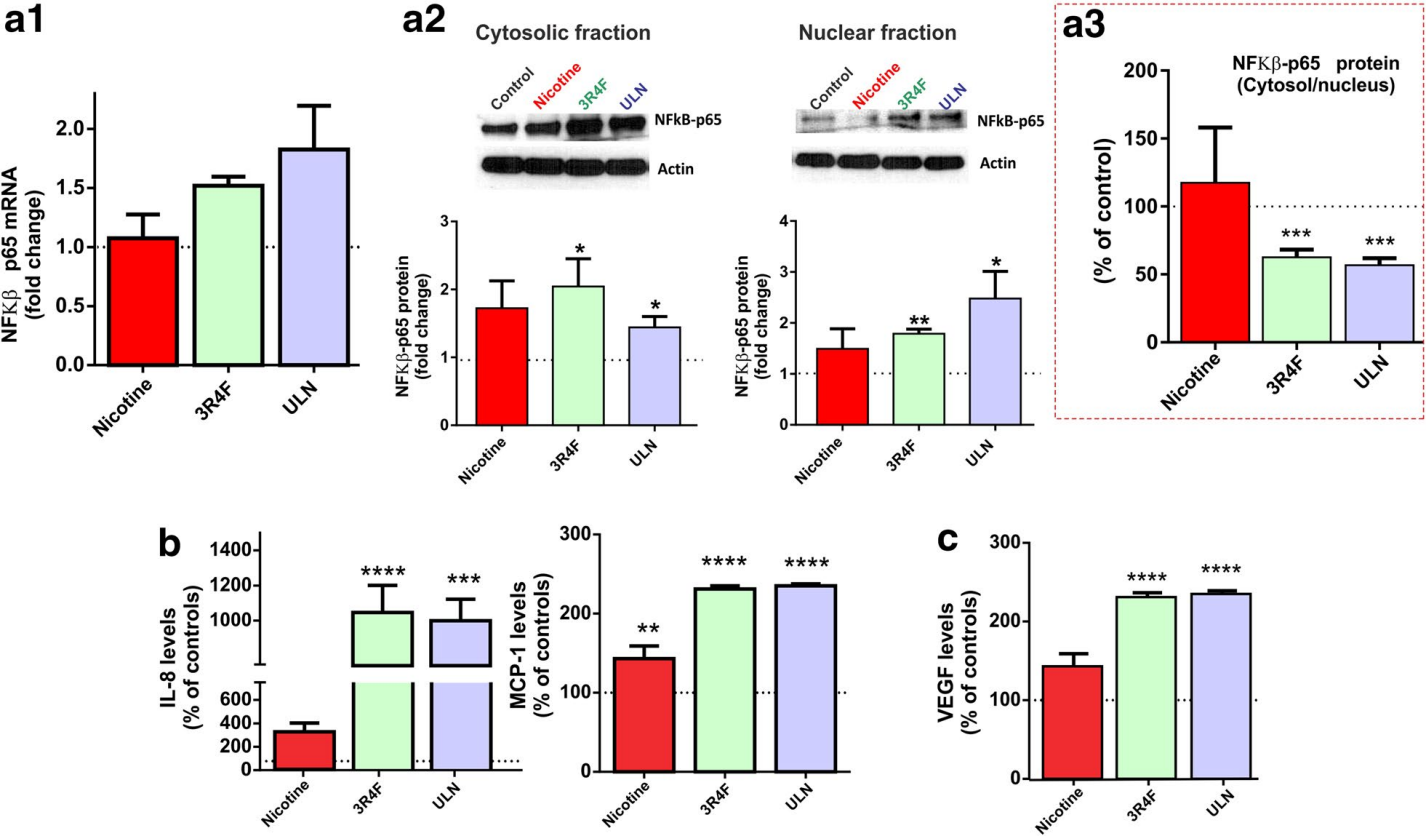
CSE exposure of various TS products induces inflammatory stress in BBB ECs. **a1** mRNA expression of NFκB-p65 in hCMEC/D3 cells following exposure to nicotine, CSE from both tobacco products; **a2** western blot analysis of cytosolic and nuclear fractions of NFκB-p65 showed increased protein expression and nuclear localization following CSEs; representative western blots were shown with actin as a loading control; **a3** the translocation status could be better understood via ratio of cytosolic versus nuclear NFκB-p65 plotted in terms of percent control; **b** release of chemokines such as IL-8 and MCP-1 from hCMEC/D3 cell cultures as determined by ELISA; **c** VEGF release was up-regulated in endothelial cultures exposed to full flavor and ULN CSE but not nicotine. Data were expressed as mean ± SEM (fold change over control). *p < 0.05, **p < 0.01, ***p < 0.001, ****p < 0.0001 vs. control. n = 6 biological replicates/condition.

We next determined the impact of TS and nicotine on the p65 subunit of NFκB (NFκB-p65) gene expression, a transcriptional factor and potential activator of oxidative and inflammatory stress pathways [22]. As shown in Figure 7a1, the mRNA expression of NFκB-p65 was markedly elevated by CSE exposure of both products with ULN producing a stronger response in 3R4F. We also observed a significant increase in the activation and nuclear translocation of NFκB-p65 following exposure to either 3R4F or ULN smoke extracts (Figure 7a2). As shown in Figure 7a3, the cytoplasm/nuclear ratio of NFκB-p65 in BBB endothelial cultures exposed to 3R4F or ULN smoke extracts was only 50% of that measured in controls. By contrast, nicotine exposure alone did altered neither the mRNA expression nor the nuclear translocation of NFκB-p65 (Figure 7a1–a3).

Secretion of the pro-inflammatory cytokine interleukin 8 (IL-8) and the chemokine Monocyte Chemoattractant Protein-1 (MCP-1) are in agreement with NFκB-p65 activation (Figure 7b). Both CSE from 3R4F and ULN cigarette products amplified the IL-8 secretory response by tenfold compared to control cultures (Figure 7b, p < 0.0001). Nicotine (100 ng/mL) was also found to significantly elevate the release of IL-8 from BBB endothelial cells (>2-fold), although the magnitude of nicotine’s effects was relatively very low when compared to CSEs (Figure 7b). In addition, release of vascular endothelial growth factor (VEGF), a known modulator of vascular angiogenesis, was also significantly increased in response to the exposure to 3R4F and ULN-derived CSE (Figure 7c, p < 0.0001). Nicotine treatment alone was also found to induce the endothelial secretion of MCP-1 and VEGF by a marginal 1.5 fold over control (Figure 7b, c).

## Discussion

The role of Nrf2 dependent antioxidant response pathway is well established in disorders aggravated by cigarette smoke such as lung emphysema, COPD, atherosclerosis and cardiac dysfunction [23–25]. In these disorders Nrf2 deficiency has clearly been demonstrated to increase the susceptibility to cellular toxicity [26] and several natural and/or synthetic antioxidant based supplemental therapies have proven to be beneficial in ameliorating the oxidative damage [27, 28].

In this study we assessed the activity and biological responses of the human BBB endothelium to a number of biological factors related to Nrf2 activation. The endpoint goal of this work was to unravel (at a molecular level) the cause–effect relationship between the oxidative potential of full flavor (3R4F) and “reduced exposure” (ULN) tobacco products and the biological response of these cells.

As expected the biological response of the BBB endothelium in respect to Nrf2 expression/activity supported our original hypothesis of a direct correlation between the oxidative potential of a tobacco product and its cellular toxicity. This is clearly shown in Figure 1 demonstrating a striking relationship between oxidative stress generated by the CSE extracts on cultured endothelial cells (Figure 1a) and the effect on the expression level and nuclear translocation of Nrf2 in the same cells (Figure 1d). A data that already argue with the notion of “reduced exposure” tobacco product (such as ULN) being intrinsically less harmful than conventional cigarettes (such as 3R4F). Our results also indicate that cigarette reduction in nicotine content has a negligible or at best a very modest effect on the overall “safety” of the product itself in terms of oxidative damage to cells and tissues.

Nrf2-dependent anti-oxidant response is mainly divided into detoxification enzymes (such as Phase I—oxidation/reduction, Phase II—conjugation enzymes and Phase III—efflux transporters) and anti-oxidant based system (such as glutathione and thioredoxin) [11]. As a follow up study, we performed transcriptome analysis to screen for the expression of several downstream targets of Nrf2-dependent antioxidant response. Nrf2 dependent downstream genes were also highly up-regulated in response to CSE exposure whereas nicotine did not bear any significant effect. Specifically, a number of Phase I detoxification genes depending upon Nrf2/ARE pathway activation were up-regulated including NQO1 (see also Additional file 1: Table S1). NQO1 is a cytoplasmic 2-electron reductase that prevents the reduction of quinones and the resulting production of radical species. NQO1 response to CSE exposure was observed at the gene expression, transcription and protein expression levels (see Figure 2). Taken together, we show that the both conventional and “reduced exposure” tobacco products (but not nicotine) elicit a strong oxidative stress response in BBB endothelial cells that well correlate with Nrf2 activation also elicited by CSE exposure.

Expression of Phase I Cytochrome P450s genes such as CYP2S1 and CYP51A1 were also up-regulated by exposure to CSE from both cigarette products although a corresponding increase in protein expression was not evident (see Figure 3). This could be possibly due to a longer time requirement for the gene expression changes to translate into measurable alteration of protein expression. Important to note however, is that very few studies have reported strong baseline expression of CYPs in normal BBB endothelium [29]. Thus, a distinct pattern of gene up-regulation of both CYP2S1 and CYP51A1 which have been observed in response to TS detoxification is a relevant piece of information further supporting the involvement of Nrf2 -dependent cytoprotection in response to TS exposure (see also Additional file 1: Table S2).

Another important component of BBB is the class of ABC transporters that function to efflux out both endogenous and exogenous toxicants out of the brain. As shown in Figure 4a, our data provides novel evidence of a potential augmentation of two classical ABCs, ABCA1 (P-gp) and ABCC4 (MRP4) at the BBB in response to CSE exposure. These changes were tangible enough to show at the gene expression level however were too small to be detected to a significant level of confidence at the transcription and translation level. Nevertheless functional assessment of an assessment of functional efflux activity specifically for P-gp (measured via Rhodamine-123; see also Figure 4c) clearly demonstrated statistically significant higher efflux function in EC cultures exposed to 3R4F. ULN effect was more modest. Considering that nicotine mildly down-regulated P-gp gene expression and did not affect its efflux activity, the difference between 3R4F and ULN in respect to P-gp activity can possibly reflect a difference in cigarette composition and substances released in solution which may differently affect P-gp expression. This provided a reasonable explanation (although difficult to ascertain given the complexity of cigarette composition) where again the total oxidative capacity of the TS product was the determining factor of cellular toxicity and activation of cytoprotective mechanisms. In addition to these major drug efflux transporters, gene array screening revealed up-regulation of several other ABC transporters primarily involved in cholesterol trafficking across the BBB (See also Additional file 1: Table S3).

The next level of cytoprotection provided by Nrf2-ARE pathway is the heightened synthesis of anti-oxidants such as GSH, thioredoxin. Exposure to CSE from 3R4F and ULN both elicited gene transcription and increase in the protein expression levels of the Cystine/glutamate transporter antiporter SLC7A11 and the Glutamate cysteine ligase regulatory subunit (also known as gamma-glutamyl cysteine synthetase) which is the first rate limiting enzyme of glutathione (GSH) synthesis (Figure 5a–c). Note, in fact, how the GSH//GSSG rate reflects the level of oxidative stress caused by TS exposure leading to GSH depletion (most evident in BBB endothelial cultures exposed to 3R4F; see Figure 5d). Note also the nicotine is capable in some measure to elicit oxidative stress. This is also reflected in the more modest but still significant decrease of GSH/GSSG ratio observed in BBB endothelial cells exposed to nicotine. Although the effect is relatively modest, this can explain while 3R4F exposure determined the most significant impact in terms of GSH reduction when compared to ULN product. ULN contain similar amount of TAR but negligible amount of nicotine. In addition, concomitant up-regulation of several other genes related to NAPDH production were also observed. (See Additional file 1: Table S4). These genes function to recycle and regenerate the anti-oxidants back to the inactive state [11].

Oxidative and inflammatory changes following TS exposure have been reported to elevate stress inducible enzyme HMOX-1 [30, 31]. Both 3R4F and ULN products showed comparable alterations in HMOX-1 (see Figure 6) and related genes (such as pirin, ferritin). Interestingly, apart from metal chelation; pirin is also reported to govern the activation of NFκβ and its related genes via Nrf2 [21]. It is a non heme Fe protein that may function to sense redox stress for the NFκβ pro-inflammatory signalling and govern the expression of downstream genes involved in immune responses [21, 32, 33]. Ferritin instead is a major intracellular storage protein which helps with iron homeostasis. It is usually composed of various ratios of light chain and heavy chain subunits. Following CSE treatment we observed increased transcription of several of these subunits whereas, both 3R4F and ULN products led to the activation of genes related to iron sequestration. In contrast, nicotine failed to induce any changes in gene and/or protein expression of HMOX-1. This again reiterated that the level of BBB toxicity was dependent on the oxidative and/or inflammatory capacity of the TS product which may not be discriminated by the nicotine content alone.

Apart from Nrf2, another cell survival transcription factor reported in event of oxidative stress is nuclear factor kappa-light-chain-enhancer of activated B cells (NF-κB); a protein complex that controls DNA transcription and is involved in cellular responses to harmful stimuli (including free radicals, pro-inflammatory cytokines, ultraviolet irradiation, etc.) and plays a key role in regulating the immune and cell survival [22]. NFκB-p65 not only increased in total cytosolic levels, but also showed increased translocation into the nucleus (See Figure 7) in response to CSE exposure. Apart from classical activation of NFκβ in events of inflammatory/oxidative stress, Nrf1/Nrf2 can up-regulate GCLC transcription indirectly via modulation of levels of NFκβ [34, 35]. Although based on current results we cannot assertively state if NFκB-p65 was directly or indirectly activated; we observed a significant increase of its nuclear translocation following CSE exposure. The effect was particularly enhanced in response to ULN-derived CSE; a cigarette product considered “reduced exposure” and less harmful than conventional products. In addition, NFκβ-p65 expression and translocation patterns consistently paralleled stress fighting responses as reported in earlier observations. NFκβ activation typically involves either canonical or non-canonical pathways. The selection dependents on whether p50/p65 (RelA) (canonical) or p52/RelB (non-canonical) is involved in the translocation and downstream activation. We could clearly observe increased translocation of the p65 sub unit into the nucleus indicating activation of the canonical pathway and associated pro-inflammatory/cell survival responses. At this point, it is difficult assert if only this pathway was associated with the TS toxicity coping mechanisms; further studies will be required in future to specifically address this conundrum. Furthermore, we observed an increase of IL-8 release following both 3R4F and ULN-derived CSE exposures. In agreement with our findings, p65 sub unit was reported to activate IL-8 expression via promoter binding [36].

Interestingly, previous in vitro studies by Barr et al. [37] have shown a dose dependent nicotine-induced activation of stress-dependent NFkB pathway in mesencephalic cells. However, we observed only a marginal response to nicotine exposure (including NFκβ-p65 protein expression and nuclear translocation). A possible explanation of this discrepancy could be attributed to two factors: (1) for our experiments the reference concentration of nicotine used was approximately 0.6 µM which falls to the lower end of the concentrations tested by Barr and co-workers; (2) time of assessment. In our study, oxidative stress measurements (as shown in Figure 1) were measured at 3 h at which point we did note a slight increase of oxidative stress in nicotine-exposed cultures versus controls. However, measurements of downstream effects such as NFkB-p65 activation (expression and nuclear translocation) were assessed at 24 h post exposure. It is possible that the oxidative stress effects elicited by nicotine fade off and became negligible at that time point.

As a part of vascular inflammation; cigarette-toxicity can lead to the up-regulation of several chemokines that promote cellular adhesion of white blood cells and facilitate their extravasation across the brain vasculature [38]. To understand the extent of vascular inflammation produced in the ECs following CSE exposure; we measured two major chemokines (IL-8 and MCP-1). Both these chemokines have been reported to be involved in the chemotaxis of neutrophils and monocytes after cigarette smoke exposure in lung and other peripheral vasculatures [39–41]. Release of IL-8 and MCP-1 was significantly increased in BBB endothelial cultures exposed to CSE (both 3R4F and ULN). This is in agreement with earlier published results by our group demonstrating a number of inflammatory changes (including IL-6 release and up-regulation of vascular adhesion molecules such as VCAM-1, Pecam-1) in BBB ECs exposed to TS [8]. Finally, CSE from both 3R4F and ULN elicited endothelial release of VEGF, a known modulator of vascular angiogenesis released in response to hypoxic condition and implicated in the alteration of BBB vascular integrity [42, 43].

## Conclusion

In summary, this novel study provides a detailed understanding of Nrf2- related cytoprotective mechanisms activated in response to TS exposure and effectively correlate them with their oxidative and inflammatory potential. Whole soluble components present in CSE and not nicotine seems to be the primary determinant of vascular toxicity. However, the identity of these specific compounds remains still elusive. Given the extremely large number of compounds contained in cigarette smoke, the identification of the specific toxicant(s) (not necessarily related to cancer genesis) responsible for the effects noted above (including potential synergistic and additive interactions) will be extraordinarily difficult to assess. In respect to our results as well as previous studies by our group and others, it is plausible that the oxidative stress stimuli generated by chronic TS exposure can effectively overwhelm the Nrf2 and NFκβ-dependent cytoprotective endothelial mechanisms. This can ultimately lead to impairment of BBB function and integrity thus promoting cerebrovascular and CNS disorders. It is in fact well known that (1) chronic smokers suffer from antioxidant shortage (e.g., vitamin C and E) caused by increased anti-oxidative mobilization in response to systemic oxidative stress evoked by ROS-enriched TS [44, 45]; (2) antioxidant supplementation reduces the TS-dependent oxidation and inflammation in animals and cells [10, 46]; (3) cigarette smoking facilitate the development of a pro-atherosclerotic environment by inducing inflammation (recruitment of leukocytes through cytokine signaling [38–41, 47] and oxidation of low-density lipoprotein cholesterol [7, 48]. Additional in vivo studies using direct inhalation models will be necessary to reveal the extent of the damage and risks associated with chronic tobacco smoking at the cerebrovascular level.

## Methods

### Materials and reagents

The antibodies used in this study were obtained from the following sources: Rabbit Nrf2 (#ab62352) from Abcam; rabbit MRP-4 (#12705S), mouse NQO1 (#3187), rabbit SLC7A11#12691, rabbit HMOX-1 #5061, NFkb p65 #8242 from Cell Signaling Technology (Danvers, MA, USA); mouse GCLC #WH0002729M1, mouse GCLM #WH0002730M, rabbit CYP2S1 #SAB4501343, rabbit CYP51A1 (#C8873), β-actin #A5441 from Sigma-Aldrich (St. Louis, MO, USA); Mouse P-gp from Calbiochem (#517310); donkey anti-rabbit (#NA934) and sheep anti-mouse (#NA931) HRP-linked secondary antibodies from GE Healthcare (Piscataway, NJ, USA); mouse anti-claudin 5 (#35-2500), goat anti-rabbit (#A11008) and anti-mouse (#A21422) conjugated to Alexa Fluor^®^ 488 and 555 from Invitrogen (Camarillo, CA, USA). Sterile culture ware was obtained from Fisher Scientific (Pittsburgh, PA, USA), while other reagents and chemicals were purchased from Sigma-Aldrich (St. Louis, MO, USA) or Bio-rad laboratories (Hercules, CA, USA).

### TS preparation

We followed a standardized ISO/FTC standard smoking protocol (35 mL draw, 2 s puff duration, 1 puff per 60 s), using a Single Cigarette Smoking Machine (SCSM, CH Technologies Inc., Westwood, NJ, USA) to prepare our cigarette smoke extracts (CSE) as previously detailed elsewhere [8]. 8 puffs per cigarette were bubbled directly into phosphate buffered saline (PBS). Three cigarettes per preparation were used to make 3× concentration (or 300%) stock solution which was then diluted to the desired concentration. Two types of cigarettes were used for this study: (a) full flavor cigarettes (3R4F, University of Kentucky) equivalent to conventional full flavor brands containing 9.4 mg tar and 0.726 mg nicotine per cigarette; (b) ultralow nicotine Spectrum cigarettes (from NIH/NIDA) equivalent to ultralow nicotine brands containing 0.03 mg nicotine and 9 mg tar per cigarette. Tar content of these cigarettes is similar to that of 3R4F (full flavor product) but that of nicotine is negligible.

### Cell culture

The hCMEC/D3 cells donated by Dr. Couraud (INSERM, Paris) (passage 29–30) were seeded on collagen coated cell culture flasks (2.0 × 10^4^/cm^2^) and maintained at 37°C with 5% CO_2_ in EBM-2 basal medium (Lonza, Walkersville, MD, USA) supplemented with 5% FBS (Atlanta Biologicals, Lawrenceville, GA, USA), Fibroblast Growth Factor (Sigma Aldrich Inc), chemically defined lipid concentrate (Life Technologies, Carlsbad, CA, USA), antibiotic/antimycotic (1:1, Atlanta Biologicals, GA, USA and HEPES (10 mM). Medium was changed once after 2 days and then every other day until cells formed a continuous confluent monolayer. BBB endothelial monolayers were then chronically exposed (3 times/24 h) to a final 5% CSE concentration [8] derived from freshly prepared smoke extracts diluted in EBM-2 culture media. Following CSE exposure cells were processed for RNA and protein collection. Please note that a 5% CSE final concentration (using 3R4F cigarettes as reference) yields a nicotine output of ≈100 ng/mL, which is comparable to the steady state blood nicotine concentration measured in an average chronic smoker (≈20 cigarettes/day) [8, 49–51]. Experiments related to nicotine exposure as a standalone agent where performed by directly diluting nicotine in the culture media at the desired concentration (100 ng/mL).

### Microarray analysis

Total RNA was extracted using RNAeasy kit (Qiagen #74104) according to manufacturer’s instructions. The RNA concentration and purity was determined using Nanodrop ND-1000, before sending the samples Boston Children’s Hospital for further microarray analysis. A total of 100 ng of RNA was required for the hybridization along with appropriate quality control (QC) samples. Illumina Human HT-12 expression bead chip was utilized for the hybridization of samples on the chip (a total of 12 samples per chip). Raw data in the form of signal intensities for each gene probe were screened, filtered and converted into fold change values over control.

### Real-time PCR

RNeasy mini kit (Qiagen Inc, #74104) was used for the extraction of total RNA from BBB endothelial cell cultures. Briefly, cells were lysed using RLT buffer and homogenized using a QIA shredder. The total RNA was then extracted from the lysate as per the manufacturer’s guidelines. Further, samples were analyzed by Nanodrop ND-1000 for quantity and purity (260/280 ratio close to 1.9–2.1). A 20-μL first-strand cDNA synthesis reaction was performed using SuperScript III (Life Technologies, carlsbad, CA) and 5 μg of total RNA. 1μL of resulting cDNA was used in a PCR reaction of 25 μL containing 2 μL of each primer (10 μM). Primer pairs used in amplification have been detailed out in Additional file 1: Table S5. Amplification was performed using a Bio-Rad CFX96 Touch™ Real-Time PCR detection system. The relative expression changes of the target genes i was then determined by ΔΔCt method [52], wherein target gene expression (based on Ct) was normalized against the house keeping gene (β-actin).

### Western blotting

Proteins was collected and fractionated using Subcellular Protein Fractionation Kit for cultured cells (Thermo scientific, #78840) according to the manufacturer’s guidelines. Protein was quantified using Pierce BCA Protein Assay Kit (Thermo Scientific, #23225) and then denatured at 95°C for 5 min using sample buffer. Denatured samples (15–20 μg) were subjected to SDS-PAGE (4–15% gradient gel) and then transferred to PVDF membranes (2 h transfer at 100 V) for further blotting. These PVDF membranes were blocked for 2 h using 5% non-fat dry milk in Tris buffered saline (TBS) containing 0.1% Tween-20 (TTBS) and subsequently incubated overnight with mouse (1:500) or rabbit (1:1,000) primary antibodies. This was followed by four TTBS washes (10 min each) and subsequent incubation with anti-rabbit or anti-mouse (1:10,000) HRP-conjugated secondary antibodies for 2 h. After four TTBS washes of 10 min each, the blots were developed using chemiluminescence detection method (using X-ray film based autoradiography). Membranes were subsequently stripped and probed for other proteins. Ms β-actin (1:10,000) was used as a loading control. Band densities were analyzed by Licor Software, normalized to actin and expressed as fold change over control protein expression.

### Immunofluorescence analysis

Cells were cultured in a two-well chamber slides for IFC analysis. Cells were fixed with 4% formaldehyde (15 min at 4°C) after period of 24 h. The slides were then washed with PBS, 5 min each for a total of three washes. This was followed by blocking with 5% goat serum (Sigma-Aldrich, St. Louis, MO, USA) in PBS at room temperature for 50 min. Fixed cells were then incubated with primary antibodies (prepared in 5% GSA in PBS) at 4°C overnight. After PBS washes, cells were incubated for 1 h at RT with Alexa Fluor^®^ 488 conjugated goat anti-rabbit or Alexa Fluor^®^ 555 conjugated goat anti-mouse secondary (1:1000) antibodies. After three washes with PBS (of 5 min each), cells were rinsed, air dried and mounted with DAPI in Prolonged Gold Anti-fade mounting media (Invitrogen, OR, USA). They were left for overnight drying in the dark before imaging with EVOS digital inverted fluorescence microscope. Cells stained with secondary antibodies alone were used as negative controls.

### GSH/GSSG analysis

GSH/GSSG ratio in BBB endothelial cell cultures was determined using GSH/GSSG Ratio Detection Assay Kit (Fluorometric-Green) (Abcam Inc. #ab138881) according to the manufacturer’s guidelines. The samples were prepared by lysis of total cell protein in T-PER lysis buffer followed by a dilution of 1:50 for GSH analysis. In brief, serial dilution of GSH and GSSG stock standards were prepared along with assay mixtures for detection of GSH and total GSH using 100× Thiol green stock solutions, assay buffer and GSSG probe. A one- step fluorimetric reaction of sample with respective assay buffers were incubated for 30 min. Fluorescence intensity was then monitored at Ex/Em of 490/520 nm. GSSG was determined by subtracting GSH from total GSH. Finally ratio of GSH was plotted against GSSG to obtain the GSH activity.

### Statistical analyses

Data from microarray as well as proteomic studies were expressed as fold changes over control ± standard error of mean (SEM). The data from all four groups were analyzed by *t* test or one-way ANOVA using GraphPad Prism Software Inc. (La Jolla, CA, USA). Post hoc multiple comparisons were conducted using Tukey’s test. P values ≤0.05 were considered statistically significant.

## Additional file

**Additional file 1:** Please refer to SI for primer sequences used for qRT-PCR and additional tables related to gene expression studies.
