# Supplementary material for: Effect of full flavor and denicotinized cigarettes exposure on the brain microvascular endothelium: a microarray-based gene expression study using a human immortalized BBB endothelial cell line
Source: BMC Neurosci. 2015 Jun 23;16:38. doi: 10.1186/s12868-015-0173-3 (PMC4477310; doi:10.1186/s12868-015-0173-3)
Supplement: Supplementary file 1 — Additional file 1: Please refer to SI for primer sequences used for qRT-PCR and additional tables related to gene expression studies. [file 12868_2015_173_MOESM1_ESM.pdf]

Table S1: Phase I Detoxification genes

Gene Short name	Gene Denomination	Fold Change over control	SE	p value	Fold Change over control	SE	p value	Fold Change over control	SE	p value
		Nicotine			3R4F (Full Flavor)			Ultralow Nicotine		
NQO2	NAD(P)H Dehydrogenase Quinone 2	1.08	0.037	ns	1.18	0.026	*	1.28	0.0471	***
AKR1B1	Aldo keto reductase family 1, member B1 (aldose reductase)	1.00	0.032	ns	1.23	0.023	**	1.09	0.061	ns
AKR1C3	Aldo-keto reductase family 1, member C3 (3-alpha hydroxysteroid dehydrogenase, type II)	0.78	0.074	ns	1.65	0.086	ns	1.79	0.116	***
CBR4	Carbonyl reductase 4	0.93	0.111	ns	1.42	0.114	*	1.27	0.055	ns

Table S1: Effect of CSE on gene array studies of other genes belonging to Phase I detoxification such as NQO2, AKR1B1, AKR1C3 and CBR4. Data were expressed as mean ± SEM (fold change over control). *p<0.05, **p<0.01, ***p<0.001 vs. control. n=6

Table S2: Phase II Detoxification genes

Gene Short name	Gene Denomination	Fold Change over control	SE	p value	Fold Change over control	SE	p value	Fold Change over control	SE	p value
		Nicotine			3R4F (Full Flavor)			Ultralow Nicotine		
GSTA4	Homo sapiens glutathione S-transferase A4	0.89	0.057	ns	1.36	0.076	*	1.44	0.131	**
GSTM2	Homo sapiens glutathione S-transferase M2 (muscle)	0.94	0.038	ns	1.26	0.041	**	1.27	0.041	***

Table S2: Effect of CSE on gene array studies of other genes belonging to Phase II detoxification related genes such as GSTA1 and GSTM2. Data were expressed as mean ± SEM (fold change over control). *p<0.05, **p<0.01, ***p<0.001 vs. control. n=6

Table S3: Genes Involved in ABC Efflux Transporters

Gene Short name	Gene Denomination	Fold Change over control	SE	p value	Fold Change over control	SE	p value	Fold Change over control	SE	p value
		Nicotine			3R4F (Full Flavor)			Ultralow Nicotine		
ABCA1	ATP-binding cassette, sub-family A (ABC1), member 1	0.99	0.102	ns	1.33	0.094	*	1.12	0.043	ns
ABCA3	ATP-binding cassette, sub-family A (ABC1), member 3	0.85	0.106	ns	1.20	0.063	*	1.04	0.061	ns
ABCB6	ATP-binding cassette, sub-family B (MDR/TAP), member 6 (ABCB6), nuclear gene encoding mitochondrial protein	0.88	0.065	ns	1.31	0.094	*	1.27	0.019	*
ABCG1	ATP-binding cassette, sub-family G (WHITE), member 1 (ABCG1), transcript variant 2	0.97	0.067	ns	1.27	0.065	*	1.15	0.035	ns

Table S3: Effect of CSE on gene array studies of other genes belonging to ABC efflux transporters involved in cholesterol efflux. Data were expressed as mean ± SEM (fold change over control). *p<0.05 vs. control. n=6

Table S4: Genes Involved in NADPH Production

Gene Short name	Gene Denomination	Fold Change over control	SE	p value	Fold Change over control	SE	p value	Fold Change over control	SE	p value
		Nicotine			3R4F (Full Flavor)			Ultralow Nicotine		
IDH1	Isocitrate dehydrogenase 1 (NADP+), soluble	0.98	0.074	ns	1.44	0.037	**	1.25	0.104	ns
ME1	Malicenzyme 1, NADP(+)-dependent, cytosolic	1.01	0.180	ns	1.62	0.143	*	1.59	0.120	*
TKT	Transketolase (Wernicke-Korsakoff syndrome) (TKT)	0.90	0.061	ns	1.47	0.082	***	1.28	0.077	*
UGDH	UDP-glucose dehydrogenase	1.02	0.146	ns	1.48	0.109	*	1.43	0.066	*

Table S4: Effect of CSE on gene array studies of other genes belonging to NADPH production. Data were expressed as mean ± SEM (fold change over control). *p<0.05, **p<0.01, ***p<0.001 vs. control. n=6

Table S5: Primer sequences used in real time qRT-PCR

Target gene	Forward	Reverse
Nrf2	CGGTATGCAACAGGACATTG	AGGATGCTGCTGAAGGAATC
NQO1	TGATCGTACTGGCTCACTCA	GTCAGTTGAGGTTCTAAGAC
P-gp (ABCB1)	GCT CCT GAC TAT GCC AAA GC	TCT TCA CCT CCA GGC TCA GT
MRP-4 (ABCC4)	TGA TGA GCC GTA TGT TTT GC	CTT CGG AAC GGA CTT GAC AT
CYP2S1	ACCCCCAACCACTTGTCCACACA	TGAGGGTCCTCGTATGTCCAAAG
CYP51A1	CTACAGTCGCCTGACAACAC	CCACTTTCTCCCCAACTCTC
SLC7A11	GGTGGTGTGTTTGCTGTC	GCTGGTAGAGGAGTGTGC
GCLC	AAGCCATTCACTCCAGATTTTACC	ACAACAAACTTCAACGCAAAGC
GCLM	ACTGACTTAGGAGCATAACTTACC	AAGAATATCTGCCTCAATGACACC
HO-1	CCAGCCATGCAGCACTATGT	AGCCCTACAGCAACTGTCGC
NFkB p65	ACT GTT CCC CCT CAT CTT CC	TGG TCC TGT GTA GCC ATT GA
β-actin	CTGGCCCGGACCTGACAGA	GCCGCAGTGGCCATCTCTT

Table S5: This includes Forward and reverse primer sequences (5' -3') used in real time qRT-PCR
